# Gastric reactance as a marker for major perioperative complications in high-risk cardiac surgery patients undergoing cardiopulmonary bypass

**DOI:** 10.3389/fmedt.2025.1662981

**Published:** 2025-11-27

**Authors:** Maria M. Godinez-Garcia, Yazmin Guillen-Dolores, Adrian Soto-Mota, Rolando Alvarez, Edgar García, Ruben Gaitan, Carlos Sanchez, Ericka Chavez, Alonso Buitano, Ma del C. Lespron, Francisco J. Molina, Solange Gabriela Koretzky, Sergio Camacho, Antonio Gordillo-Moscoso

**Affiliations:** 1 Department of Clinical Research, Alandra Medical SAPI de CV, Mexico City, Mexico; 2 Department of Anesthesiology, National Institute of Cardiology (INCICH), Mexico City, Mexico; 3 Department of Research, Metabolic Diseases Research Unit, National Institute of Medical Sciences and Nutrition, Mexico City, Mexico; 4 Department of Cardiovascular Intensive Care, National Institute of Cardiology (INCICH), Mexico City, Mexico; 5 Department of Biomedical Engineering, Alandra Medical SAPI de CV, Mexico City, Mexico; 6 Faculty of Sciences, Department of Biomedical, Autonomous University of San Luis Potosi, San Luis Potosí, Mexico; 7 Faculty of Medicine, Department of Clinical Epidemiology, Autonomous University of San Luis Potosi, San Luis Potosí, Mexico

**Keywords:** gastric reactance, continuous monitoring, intensive care unit, cardiopulmonary bypass, perfusion markers

## Abstract

**Background:**

Gastric reactance (XL) is a bioelectrical property of the stomach lining that responds to changes in gut perfusion. It is measured through bioimpedance spectroscopy, a technology that assesses the tissue's electrical resistance and capacity to store electrical charge, providing insight into the physiological state of the gastric mucosa.

**Objective:**

This prospective observational study explored the relationship between XL and hemodynamic variables in high-risk adult patients undergoing elective cardiac surgery with cardiopulmonary bypass (CPB) at the National Institute of Cardiology, Mexico City.

**Methods:**

A binary composite endpoint was constructed to aggregate major perioperative complications into a single outcome measure. The sample size was calculated based on anticipated event rates. Associations among variables were examined using nonparametric statistical tests. Predictive performance, including confidence intervals, was estimated using bootstrapped receiver operating characteristic (ROC) curves.

**Results:**

Thirty-seven patients were enrolled and categorized according to the development of major perioperative complications (MPOC; *n* = 23) or absence thereof (Non-MPOC; *n* = 14). Baseline demographic and intraoperative variables did not differ significantly between groups. However, the MPOC group exhibited higher postoperative severity scores (APACHE II: 21.5 vs. 18.5, *p* = 0.231; SOFA: 12.5 vs. 12.0, *p* = 0.249) and greater postoperative bleeding (1.0 L vs. 0.4 L, *p* < 0.001). XL minimum values (XL_Min) were consistently elevated in the MPOC group throughout all perioperative events, with a significant shift of 6.14 -jΩ (95% CI [1.06, 11.34], *p* = 0.022) in Post-CPB.

**Conclusion:**

These findings suggest that gastric impedance spectroscopy is a safe and feasible technique for intraoperative and postoperative monitoring, and that elevated XL_Min values may aid in the early identification of patients at risk for MPOC by detecting gastric mucosal hypoperfusion during high-risk cardiac surgery.

## Introduction

Cardiac surgery patients frequently experience a range of complications, while some are common and predictable, there are Major Perioperative Complications (MPOC) such as, excessive bleeding (10%), vasoplegic syndrome (5%–25%), and cardiogenic shock (3%–8%) that present the highest risk for the patients' outcome (1–3). Such complications stem from systemic disturbances affecting multiple organs, making it critical for clinicians to possess a comprehensive understanding of the multisystem pathophysiology behind the status of the patient for timely identification and management of complications (1). To predict mortality and complications, scoring models such as the Sequential Organ Failure Assessment (SOFA), the Acute Physiology and Chronic Health Evaluation (APACHE-II), EuroSCORE II, and Society of Thoracic Surgeons (STS) risk scores are widely used. While effective, these models have specific time frames of application, i.e., SOFA and APACHE-II are most useful in the first 24 h post-surgery, while EuroSCORE II and STS scores are more relevant preoperatively (4, 5). Particularly for cardiogenic shock, the Society for Cardiovascular Angiography and Interventions (SCAI) proposed a method for classification and management that is widely used, the SCAI SHOCK stage classification stratifies patients from SCAI-A to SCAI-E (6). This method uses a 3-axis model which incorporates shock severity, clinical phenotype, and risk modifiers.

Hemodynamic monitoring is an essential aspect of managing cardiac surgery patients. Pulmonary Arterial Catheters (PAC) are useful for obtaining real-time measurements of hemodynamic variables such as Mean Arterial Pressure (MAP), Cardiac Index (CI), Pulmonary Artery Wedge Pressure (PAWP), and Mixed venous oxygen saturation (SvO_2_) (7). These parameters are key indicators of macrocirculation, which refers to the large-scale circulatory dynamics of the body. Monitoring these variables allow clinicians to maintain macrocirculatory stability by keeping their values within target ranges, crucial for patient survival (7–9); however, macrocirculatory stability does not ensure adequate perfusion to all organs (e.g., hypoperfusion during normotension) (8).

Complementing hemodynamic monitoring with other variables like metabolic markers or microcirculatory indicators gives a better understanding of mechanisms affecting perfusion. For instance, blood lactate (LCT) levels have long been used as a marker of tissue hypoperfusion, with hyperlactatemia (>3 mmol/L) indicating a higher likelihood of adverse outcomes (9). However, lactate alone is insufficient to determine circulatory failure, as it cannot account for regional variations in oxygen delivery, which may differ from global oxygen delivery (10).

Considering the above, clinicians move away from focusing on single, predefined threshold values of hemodynamic parameters and emphasize a more individualized approach based on overarching patient characteristics and contexts, which can be informed by integrating emerging technologies that expand on the hemodynamic profile (8). For example, gastric tonometry, microcirculatory monitoring, urethral photoplethysmography, mitochondrial oxygen tension, and tissue oxygen saturation, have been proposed to provide more accurate assessments of tissue perfusion (11–14).

This work focuses on gastric impedance spectroscopy (15, 16), a technique that continuously measures impedance of the gastric wall at multiple frequencies to calculate gastric reactance (XL). Measured in imaginary ohms (jΩ), this parameter senses bioelectrical properties of the gastric mucosa which reflect variations in gastrointestinal perfusion (15). Thereby, it provides physiologically relevant information about the state of the stomach lining. Since changes in tissue oxygenation cause cellular alterations that manifest as measurable changes in the tissue's electrical impedance, this parameter functions as a marker for inadequate gastric mucosa perfusion (15–18). XL is hypothesized to be a sensitive marker of mucosal ischemic injury due to splanchnic hypoperfusion in critically ill patients. This study aims to explore XL in patients likely to develop MPOC, comparing key hemodynamic variables, and metabolic markers with XL along each perioperative event in high-risk patients undergoing CPB-assisted cardiac surgery. Monitoring occurred throughout the operating room (OR), the first four hours in the ICU, after 24 h, 48 h, and at a 30-day follow-up.

## Material and methods

### Study design

**Figure 1:**
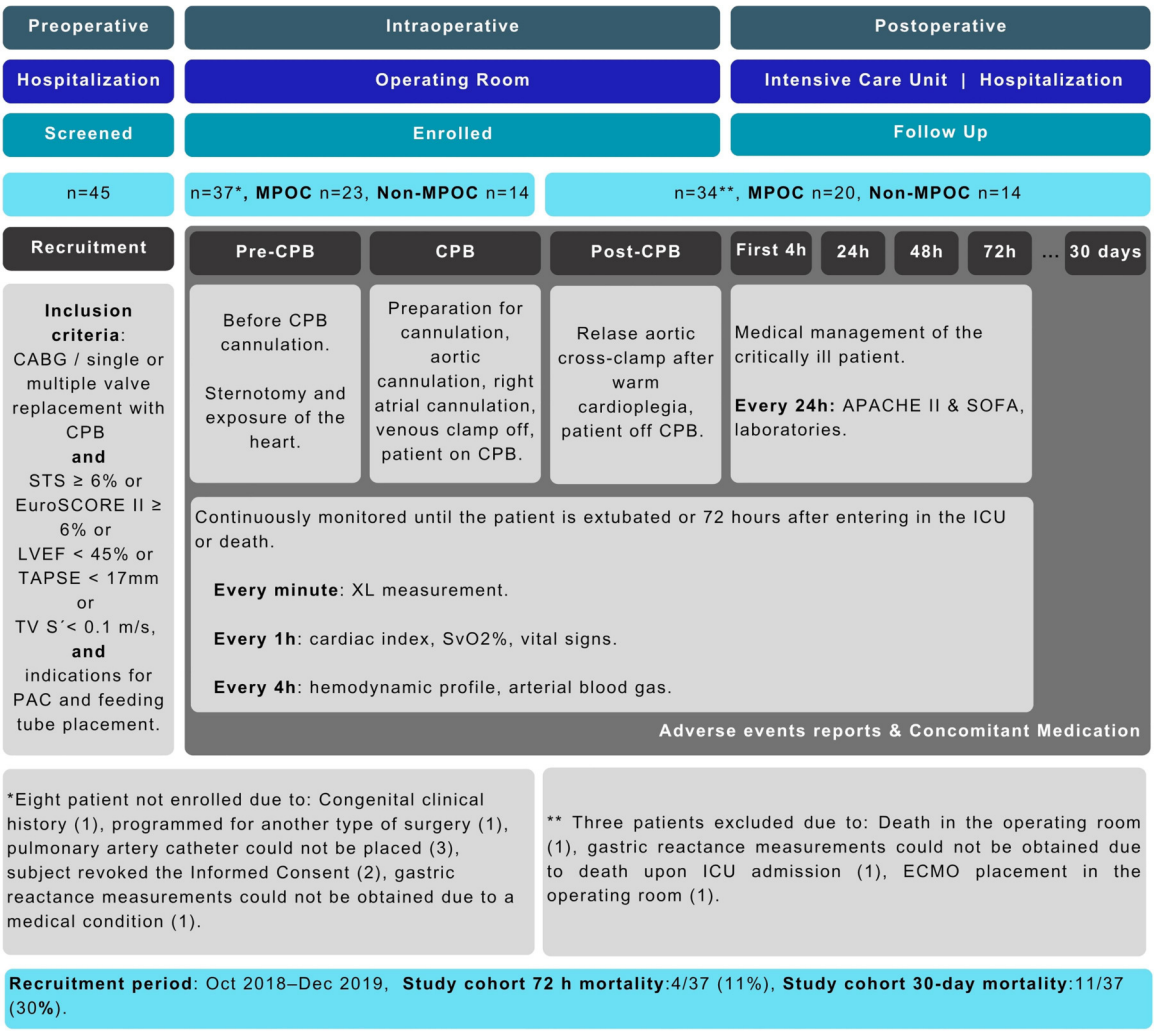
Study design and STROBE flowchart. APACHE II, acute physiology and chronic health evaluation; CABG, coronary artery bypass graft; CPB, cardiopulmonary bypass; EuroSCORE II, European system for cardiac operative risk evaluation II; ICU, intensive care unit; LVEF, left ventricular ejection fraction; MPOC, major postoperative complications; PAC, pulmonary artery catheter; STS, society of thoracic surgeons; SOFA, sequential organ failure assessment; SvO2, mixed venous oxygen saturation; TAPSE, tricuspid annular plane systolic excursion; TV S', systolic peak velocity of the lateral ring of the tricuspid; XL, gastric reactance.

This prospective cohort study analyzed measurements (gastric impedance, hemodynamic, laboratories, blood gas, vital signs, and risk scales) taken during three key phases: preoperative, intraoperative, and postoperative for patients undergoing elective cardiac CPB-assisted surgery. During the intraoperative phase, measurements were taken at three specific events: before CPB cannulation (Pre-CPB), during CPB (CPB), and after CPB (Post-CPB). In the postoperative phase, measurements were taken at five different events: the first four hours in the ICU, after 24 h, 48 h, 72 h, and at a 30-day follow-up (see Figure 1). Given the observational nature of the study, no blinding was implemented, and the XL marker values influenced no diagnostic or therapeutic decisions.

The study was conducted according to the Declaration of Helsinki, Good Clinical Practices of the International Conference of Harmonization (ICH E6 R2), ISO 14155:2011 Clinical investigation of medical devices for human subjects—Good Clinical Practice, and Mexican regulations—the General Health Law regarding Health Research, and NOM-012-SSA3-2012. The study was approved by Local Ethics Committees and authorized (193300912X0085/2019) by the Mexican Ministry of Health through the Federal Commission for the Protection against Sanitary Risks (COFEPRIS). The study was registered at ClinicalTrials.gov under registration ID NCT03799133. All study participants provided written informed consent. This study was reported by the STROBE statement for observational studies (see Supplementary Table S1). The recruitment period for this study was from October 1, 2018, to December 28, 2019 at the Intensive Care Unit in the National Institute of Cardiology (INCICH), Mexico City, Mexico.

### Inclusion and exclusion criteria

The study included men and women aged 18 years and older, in sinus rhythm, scheduled for elective cardiac procedures such as valvular replacement, revascularization, or both. Eligible participants required intraoperative monitoring with a pulmonary artery catheter (PAC) with no contraindications for feeding tube placement for measuring XL. Additionally, to ensure a high-risk profile, patients had to meet at least one of the following conditions: mortality risk score STS equal to or greater than 6%, or EuroSCORE II equal to or greater than 6%, or left ventricular ejection fraction (LVEF) less than 45%, or displacement of the tricuspid annular plane systolic excursion (TAPSE) less than 17 mm, or systolic peak velocity of the lateral ring of the tricuspid (TV S') measured by tissue Doppler imaging less than 0.1 m/s. Exclusion criteria were gastrointestinal bleeding in the past 30 days, stroke, congenital heart disease, maxillofacial malformation, inability to place a feeding tube, pregnancy, lactation, permanent pacemaker or defibrillator, or contraindication to orogastric/nasogastric tube placement.

### Clinical management

Vital signs, hemodynamics, anesthetics, and fluid management adhered to standard in-hospital and international clinical protocols. All patients underwent oral intubation, CPB, and were maintained on an anesthesia machine (Datex Ohmeda/5, General Electric). Anesthetic induction was performed using standard doses: fentanyl (3 µg/kg), etomidate (0.1–0.2 mg/kg), rocuronium (0.6 mg/kg), and lidocaine (1 mg/kg).

A central venous catheter and a pulmonary artery catheter (PAC) equipped with a SvO₂ probe were inserted into the right internal jugular vein. Hemodynamic profiles were obtained via the PAC and calculated using established equations (7, 19) during both intraoperative (pre-CPB and post-CPB) and postoperative (first 4 h in the ICU) phases. Cardiopulmonary bypass (CPB) was conducted by institutional protocols and international standards (20–22). Following surgery, all patients were transferred to the ICU. The clinical management of each patient was at the physician's discretion, reflecting the observational nature of the study. Data collection was performed using a HIPAA-compliant electronic data capture platform (Medrio™).

### Gastric impedance measurements

**Figure 2:**
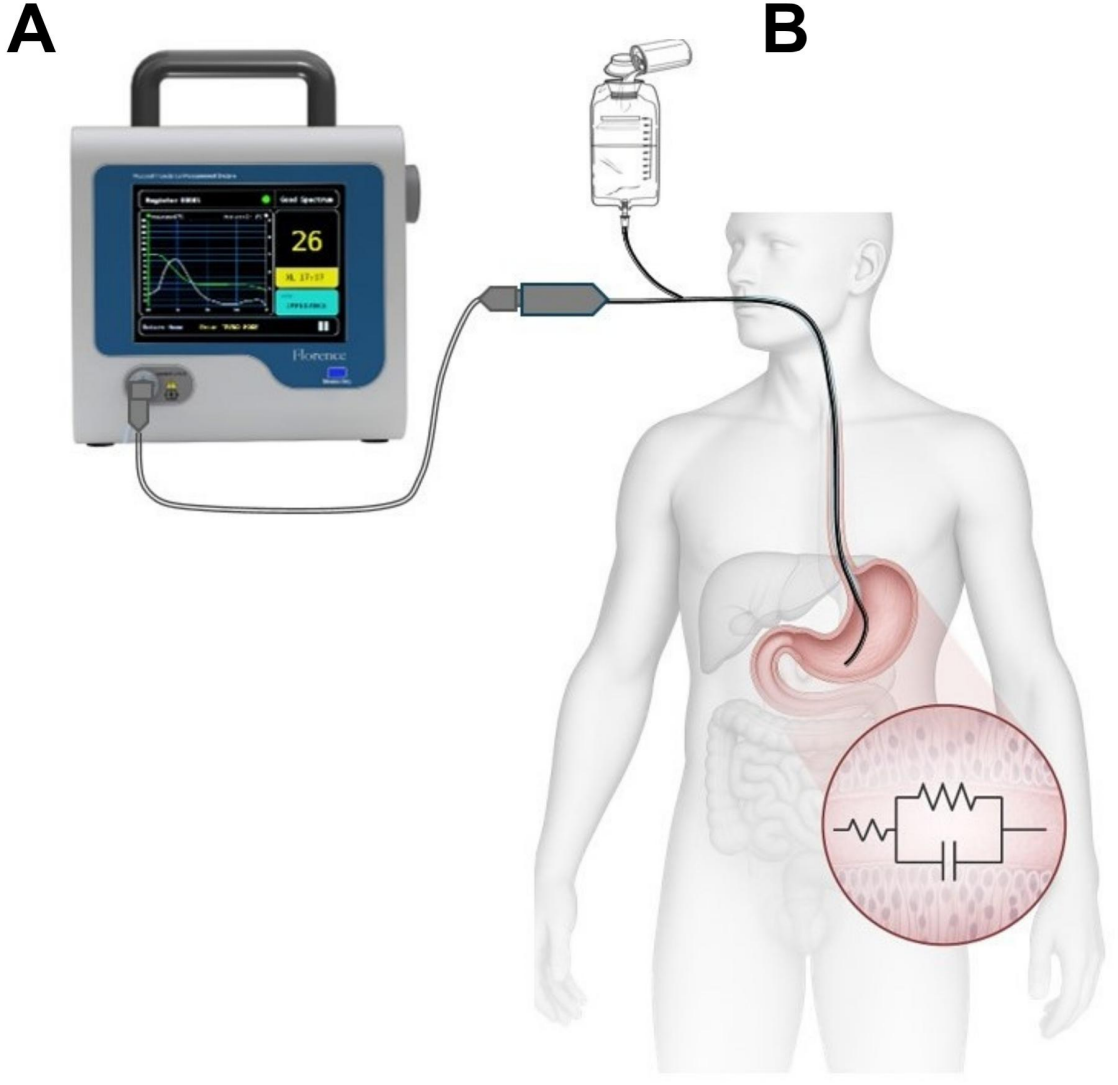
Mucosal impedance measurement system (MIMS). **(A)**—bedside monitor; **(B)**—sample applications in humans with a feeding tube fitted with an impedance sensor.

In this study, XL was measured through the Mucosal Impedance Measurement System (MIMS), which comprises a feeding tube equipped with an impedance sensor (Athena, model ISMO-16-120, Alandra Medical) and a bedside monitor (Florence, model ISMO 1.0, Alandra Medical) that displays both real-time (every min) and trend values of XL (every 10 min). To maintain optimal signal quality, gastric secretions were aspirated from the feeding tube every 20 min (see Figure 2).

### Statistical analysis

The study utilized a binary composite endpoint (CEP), which was defined as an outcome combining several endpoints of interest within a single variable (23, 24). It is important to note that the CEP does not reflect the effects of individual components, even if they individually show statistically significant and clinically relevant effects.

The CEP was defined as the occurrence of at least one of the following high-risk complications: cardiogenic shock (CI ≤ 2.2 L/min/m^2^ and PAW*P* ≥ 15 mmHg), clinically significant bleeding (801–1,000 mL) (25), vasoplegic syndrome (in clinical practice vasoplegia can be assessed clinically by the vasopressor dosage necessary to maintain MAP and by the drop in diastolic blood pressure) (26), or death. Patients who develop any CEP component were categorized into the MPOC group, while those without such complications during follow-up were included into the Non-MPOC group.

The sample size was determined using the methodology proposed by Peduzzi and Concato (27), which recommends 10–20 events per variable (EPV) for time-to-event study designs. Expected incidences were derived from published data and institutional records: 6% for cardiogenic shock (2), 10% for clinically significant bleeding (1), 5% for vasoplegic syndrome (1), and 8.4% for mortality (as reported by INCICH), yielding a cumulative CEP incidence of 29.4%. Based on these estimates, the required sample size ranged from 34–68 patients. Ultimately, 37 patients who met the eligibility criteria were included in the study.

All variables included in the analyses met a ≥85% measurement threshold. Variables below this threshold, corresponding to event-time data, were excluded and are not reported in the results (28). For all variables, minimum and maximum values were calculated for each event to increase accuracy (29). The XL minimum values (XL_Min) were chosen to consider its worst-case scenario (15). The Shapiro–Wilk test was employed to assess the normality of continuous variables; however, nonparametric methods were utilized due to the prevalence of non-normal data. Normality of continuous variables was tested through the Shapiro–Wilk test. Due to non-normality, Fisher's exact test was used for categorical data and the Wilcoxon rank-sum test for continuous data. Associations between XL_Min and macrocirculatory variables were evaluated with Kendall's tau-b. Perioperative group differences (Pre-CPB, CPB, Post-CPB, 4h-ICU) were analyzed with the Wilcoxon test, reporting Hodges–Lehmann estimates, 95% CIs, effect sizes, and exact *p*-values. Bonferroni correction was applied for multiple comparisons. All analyses were deemed significant for *p*-values < 0.05. Data analyses were performed in R 4.4.1 with RStudio 1.4.1106 (Windows 11).

The predictive performance of XL_Min for morbimortality was evaluated and compared against key variables using receiver operating characteristic (ROC) curves. Binary classification was based on the presence or absence of MPOC within the CEP cohort. The model performance was quantified by positive predictive value (PPV), negative predictive value (NPV), area under the ROC curve (AUC), sensitivity, and specificity. Furthermore, 95% confidence intervals (CIs) were estimated via 2,000 stratified bootstrap replicates to ensure robust inference.

## Results

A total of 37 subjects were enrolled in the study (8 out of 45 screened individuals were excluded for not meeting the inclusion criteria). Figure 1 illustrates the participant's workflow. During the recruitment period, 5,831 surgical procedures were performed at the institution, with an overall hospital mortality rate of 5.81%.

Within the study cohort, the 72 h mortality rate was 11% (4/37), and the 30-day mortality rate was 30% (11/37). MPOC occurred in 62% of patients (23/37), including bleeding (35%), cardiogenic shock (22%), and vasoplegic syndrome (5%). The remaining 38% (14/37) did not develop MPOC (Non-MPOC group). The mortality rate among patients with MPOC was 48% (11/23), with all deaths associated with at least one additional complication.

Intraoperative transfusion volumes were comparable between groups: the MPOC group received a median of 812.0 mL (IQR: 384.0–1,239.0), while the Non-MPOC group received 673.5 mL (IQR: 311.0–1,198.0; *p* = 0.584). At 24 h postoperatively, transfusion volumes were 392.0 mL (IQR: 272.0–752.5) in the MPOC group and 449.5 mL (IQR: 246.5–849.0) in the Non-MPOC group (*p* = 0.925).

Table 1 compares baseline demographic and clinical characteristics, revealing no statistically significant differences between the MPOC and Non-MPOC groups, confirming that both cohorts consisted of high-risk patients. Table 2 presents perioperative cardiovascular risk factors, with significant differences observed only in postoperative bleeding events (*p* = 0.001).

**Table 1:** Baseline demographic and clinical characteristics.

Variable	Overall *n* = 37	MPOC *n* = 23	Non-MPOC *n* = 14	*p*-value
Age, year	52.0 (18.0)	52.0 (15.5)	49.0 (27.5)	0.962^a^
Female gender	11 (30%)	9 (39%)	2 (14%)	0.150^b^
BMI, kg/m^2^	27.0 (3.6)	26.7 (4.2)	27.3 (3.4)	0.294^a^
Type of Surgery				0.643^b^
First surgery				
Single procedure	21 (57%)	13 (57%)	8 (57%)	
Combined procedure	11 (30%)	6 (26%)	5 (36%)	
Reoperation				
Combined procedure	5 (14%)	4 (17%)	1 (7.1%)	
Admission criteria				0.074^b^
EuroSCORE II ≥ 6%	6 (16%)	6 (26%)	0 (0%)	
LVEF < 45%	23 (62%)	14 (61%)	9 (64%)	
Combined surgical procedures	8 (22%)	3 (13%)	5 (36%)	
Diabetes	11 (30%)	8 (35%)	3 (21%)	0.477^b^
Hypertension	18 (49%)	12 (52%)	6 (43%)	0.737^b^
Smoking	12 (32%)	9 (39%)	3 (21%)	0.306^b^

Data are presented as median (IQR) or Frequency (%) as appropriate.

BMI, body mass index; CABG, coronary artery bypass graft; EuroSCORE II, European system for cardiac oRisk evaluation II; LVEF, left ventricular ejection fraction; MPOC, major postoperative complications; Single procedure: single valvular replacement or CABG, Combined procedure: double or triple valvular replacement or CABG with single valvular replacement.

a Wilcoxon rank sum test.

b Fisher's exact test.

**Table 2:** Perioperative cardiovascular risk factors by groups.

Variable	Overall *n* = 37	MPOC *n* = 23	Non-MPOC *n* = 14	*p*-value
Preoperative				
BSA, m^2^	1.8 (0.3)	1.7 (0.3)	1.8 (0.3)	0.510
STS, %	1.4 (2.5)	1.2 (2.2)	1.5 (2.3)	0.609
EuroSCORE II, %	2.6 (5.1)	3.4 (6.7)	2.2 (4.1)	0.650
Intraoperative				
CPB-Time, min	167.0 (110.0)	170.0 (124.5)	162.0 (60.3)	0.672
Aortic cross-clamp Time, min	121.0 (68.0)	121.0 (83.5)	121.5 (65.3)	0.950
Surgery-Time, min	320.0 (145.0)	360.0 (149.0)	300.5 (88.0)	0.056
Urine Output, mL/Kg/h	2.5 (1.2)	2.5 (0.9)	2.6 (1.8)	0.605
Fluid Balance, L	−0.4 (1.1)	−0.4 (1.1)	−0.3 (1.4)	0.963
Bleeding, L	0.4 (0.4)	0.5 (0.6)	0.4 (0.1)	0.064
Postoperative	*n* = 34	*n* = 20	*n* = 14	*p*-value
Admission at ICU				
APACHE II, points	21.0 (4.8)	21.5 (3.5)	18.5 (4.8)	0.231
SOFA, points	12.0 (2.8)	12.5 (2.3)	12.0 (3.0)	0.249
24 h at ICU				
LVEF, %	35 (10)	34 (10)	35 (14)	0.456
Urine Output, mL/Kg/h	1.5 (0.9)	1.3 (0.7)	1.9 (0.8)	0.302
Fluid Balance, L	1.5 (2.0)	1.7 (2.4)	1.0 (1.4)	0.111
Bleeding, L	0.6 (0.7)	1.0 (0.9)	0.4 (0.4)	0.001*
Follow up				
LOS-ICU, days	4.5 (4.8)	5.5 (5.8)	3.0 (2.8)	0.179

Data are presented as median (IQR); Wilcoxon rank sum test.

* For statistically significant results (*p* < 0.05).

APACHE II, acute physiology and chronic health evaluation; BSA, body surface area; CPB, cardiac pulmonary bypass; EuroSCORE II, European system for cardiac operative risk evaluation; ICU, intensive care unit; LOS, length of stay; LVEF, left ventricular ejection fraction; MPOC, major perioperative complications; SOFA, sequential organ failure assessment; STS, society of thoracic surgeons.

**Figure 3:**
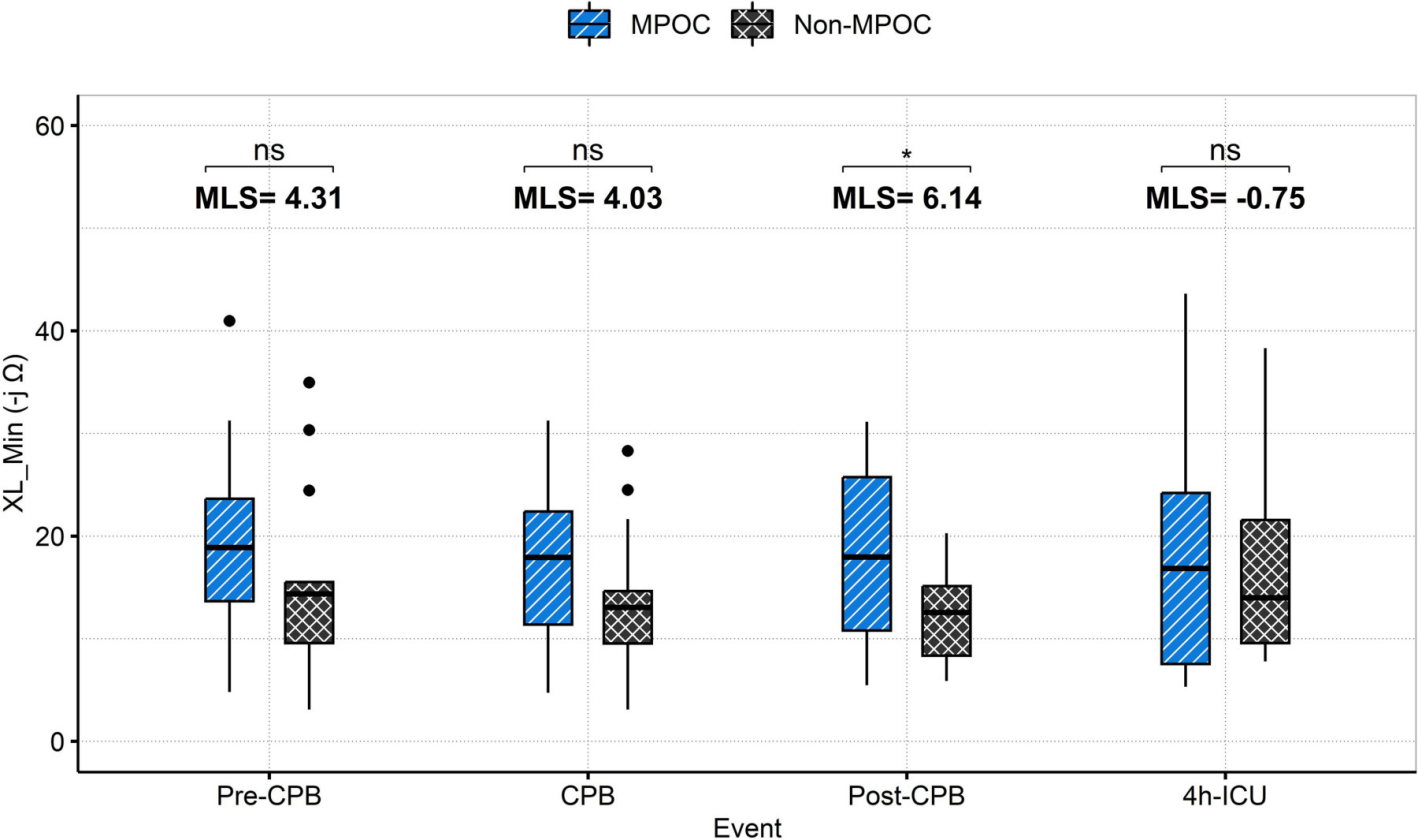
Intraoperative (pre-CPB, CPB, post-CPB) and postoperative (4h-ICU) events comparison for MPOC and Non-MPOC changes in XL_Min. *for statistically significant results (*p* < 0.05). CPB, cardiopulmonary bypass; ICU, intensive care unit; MLS, median location shift; MPOC, major perioperative complications; ns, non-significant, XL_Min, the minimum value for gastric reactance.

Figure 3 depicts the behavior of XL_Min during intraoperative and postoperative events for both groups. XL_Min values were consistently higher in the MPOC group compared to Non-MPOC, with a statistically significant difference observed during the post-CPB event.

The median location shift (MLS) analysis of XL_Min between Non-MPOC and MPOC groups across all evaluated events revealed the following (see Figure 3). During the Pre-CPB a median location shift of 4.3 -jΩ [95% CI (−1.72, 10.09), *p* = 0.115] was observed; during CPB, the shift was 4.03 -jΩ [95% CI (−1.39, 9.34), *p* = 0.122]; Post-CPB a statistically significant shift of 6.14 -jΩ [95% CI (1.06, 11.34), *p* = 0.022] was evident; and during the first 4 h in ICU, the shift was −0.75 -jΩ [95% CI (−6.17, 9.51), *p* = 0.769]. Among these, only the Post-CPB difference reached statistical significance (*p* < 0.05).

Table 3 presents the median (IQR), MLS with 95% CI [Low, High], minimum, and maximum values of key variables by event and group. SvO₂_Min was statistically significant during the pre-CPB event; XL_Min and SvO₂_Min were significant during the post-CPB event; and CI_Max and SvO₂_Max were significant during the 4-hour ICU period. Table 4 details the correlation coefficients of key variables, stratified by group and event. No statistically significant differences were observed.

**Table 3:** Intraoperative and postoperative key variables by groups and events.

	Pre-CPB				CPB				Post-CPB				4h-ICU			
Variable	MPOC *n* = 23	Non-MPOC *n* = 14	*p*-value	MLS 95% CI	MPOC *n* = 23	Non-MPOC *n* = 14	*p*-value	MLS 95% CI	MPOC *n* = 23	Non-MPOC *n* = 14	*p*-value	MLS 95% CI	MPOC *n* = 20	Non-MPOC *n* = 14	*p*-value	MLS 95% CI
XL_Min, -jΩ	18.89 (9.98)	14.36 (5.95)	0.115	4.31 (−1.72, 10.09)	17.93 (11.01)	13.06 (5.10)	0.122	4.03 (−1.39, 9.34)	17.96 (14.94)	12.55 (6.77)	0.022*	6.14 (1.06, 11.34)	16.85 (16.67)	13.99 (11.99)	0.769	−0.75 (−6.17, 9.51)
LCT_Max, mmol/L	1.40 (0.60)	1.30 (0.20)	0.276	0.10 (−0.10, 0.40)	2.20 (0.70)	2.65 (1.58)	0.594	−0.20 (−1.00, 0.50)	3.60 (3.30)	2.45 (1.40)	0.141	0.80 (−0.30, 2.40)	4.30 (4.83)	3.00 (2.33)	0.294	0.80 (−0.50, 3.00)
LCT_Min, mmol/L	1.00 (0.30)	1.15 (0.20)	0.476	−0.09 (−0.20, 0.10)	1.50 (0.50)	1.35 (0.45)	0.801	0.00 (−0.30, 0.30)	2.80 (1.95)	2.40 (0.60)	0.266	0.40 (−0.30, 1.20)	3.55 (4.10)	2.15 (1.13)	0.061	1.10 (0.00, 2.80)
MAP_Max, mmHg	74.67 (19.00)	72.17 (8.58)	0.491	3.00 (−6.00, 11.00)	-	-	-	-	67.67 (8.34)	71.00 (12.67)	0.228	−3.34 (−9.33, 2.00)	83.83 (15.25)	86.50 (10.83)	0.234	−5.66 (−14.66, 3.00)
MAP_Min, mmHg	62.00 (18.84)	58.67 (16.08)	0.616	1.90 (−6.33, 8.00)	-	-	-	-	58.33 (12.67)	63.50 (11.00)	0.069	−6.67 (−13.00, 0.66)	61.67 (10.50)	67.67 (10.92)	0.059	−6.38 (−13.33, 0.33)
CI_Max, L/min/m^2^	2.08 (0.89)	1.78 (0.94)	0.562	0.14 (−0.41, 0.69)	-	-	-	-	2.21 (0.67)	2.38 (1.54)	0.136	−0.45 (−1.14, 0.13)	2.51 (1.18)	3.29 (1.57)	0.022*	−0.90 (−1.63, −0.17)
CI_Min, L/min/m^2^	1.43 (0.65)	1.54 (0.70)	0.772	−0.06 (−0.53, 0.34)	-	-	-	-	1.90 (0.64)	2.10 (1.23)	0.146	−0.48 (−1.07, 0.11)	1.93 (0.87)	2.41 (1.39)	0.137	−0.41 (−1.05, 0.13)
PAWP_Min, mmHg	18.00 (13.00)	15.00 (3.50)	0.255	3.00 (−3.00, 8.00)	-	-	-	-	14.00 (7.00)	15.50 (5.75)	0.407	−1.66 (−5.00, 3.00)	8.50 (18.00)	10.00 (9.50)	0.531	−1.89 (−9.00, 13.00)
PAWP_Max, mmHg	22.50 (15.25)	18.50 (7.75)	0.256	4.00 (−3.00, 10.00)	-	-	-	-	18.00 (8.75)	19.50 (9.50)	0.591	−1.00 (−5.00, 4.00)	15.00 (16.50)	15.00 (9.75)	0.964	−0.92 (−10.00, 10.00)
SvO_2__Max, %	75.00 (19.50)	81.00 (14.63)	0.266	−3.85 (−13.00, 3.00)	-	-	-	-	74.00 (11.25)	83.80 (17.50)	0.128	−7.50 (−14.00, 2.00)	74.00 (13.00)	81.00 (9.00)	0.031*	−9.00 (−16.00, −1.00)
SvO_2__Min, %	69.00 (11.45)	74.00 (12.25)	0.018*	−9.00 [−16.00, −1.00)	-	-	-	-	67.00 (18.25)	79.00 (9.75)	0.012*	−9.00 (−18.00, −2.00)	69.00 (17.00)	72.00 (15.00)	0.173	−9.00 (−17.00, 3.00)

Data are presented as median (IQR); Wilcoxon rank sum test.

4h-ICU, first four hours in the intensive care unit; CI_Min, the minimum value for cardiac index; CI_Max, the maximum value for cardiac index; CPB, cardiac pulmonary bypass; LCT_Max, the maximum value for lactate; LCT_Min, the minimum value for lactate; MAP_Min, the minimum value for mean arterial pressure; MAP_Max, the maximum value for mean arterial pressure; MLS, median location shift, MPOC, major perioperative complications; PAWP, pulmonary artery wedge pressure; Pre-CPB, preoperative cardiac pulmonary bypass; Post-CPB, postoperative cardiac pulmonary bypass; SvO_2__Max, the maximum value for mixed venous oxygen saturation; SvO_2__Min, the minimum value for mixed venous oxygen saturation; XL_Min, the minimum value for gastric reactance.

* For statistically significant results (*p* < 0.05) and 95% CI (Low, High).

**Table 4:** Correlation with XL_Min.

	Pre-CPB				CPB				Post-CPB				4h-ICU			
Variables	MPOC t	*p*-value	Non-MPOC t	*p*-value	MPOC t	*p*-value	Non-MPOC t	*p*-value	MPOC t	*p*-value	Non-MPOC t	*p*-value	MPOC t	*p*-value	Non-MPOC t	*p*-value
LCT_Min, mmol/L	0.110	0.487	−0.060	0.777	−0.130	0.411	0.310	0.135	−0.040	0.791	−0.023	0.912	0.090	0.581	0.000	1.000
LCT_Max, mmol/L	0.091	0.556	0.120	0.594	−0.036	0.812	0.150	0.443	0.052	0.731	0.056	0.784	0.058	0.721	0.022	0.913
MAP_Min, mmHg	0.380	0.011	0.230	0.279	-	-	-	-	−0.240	0.107	0.330	0.100	−0.230	0.165	0.011	1.000
MAP_Max, mmHg	0.083	0.579	0.083	0.579	-	-	-	-	−0.140	0.342	−0.140	0.342	−0.120	0.455	−0.120	0.455
CI_Min, L/min/m^2^	0.026	0.866	−0.230	0.306	-	-	-	-	0.110	0.542	0.039	0.855	0.350	0.053	−0.300	0.157
CI_Max, L/min/m^2^	−0.052	0.735	−0.280	0.204	-	-	-	-	0.170	0.299	0.150	0.510	0.330	0.069	−0.055	0.830
SvO_2__Min, %	−0.250	0.091	−0.140	0.475	-	-	-	-	−0.037	0.820	−0.230	0.249	0.097	0.591	0.240	0.269
SvO_2__Max, %	−0.110	0.475	−0.089	0.660	-	-	-	-	−0.043	0.795	−0.310	0.123	0.060	0.741	0.130	0.538
STS, %	0.077	0.747	−0.210	0.548	-	-	-	-	-	-	-	-	-	-	-	-
EuroSCORE II, %	−0.230	0.140	0.055	0.830	-	-	-	-	-	-	-	-	-	-	-	-
APACHE II, points	-	-	-	-	-	-	-	-	-	-	-	-	−0.270	0.108	0.100	0.619
SOFA, points	-	-	-	-	-	-	-	-	-	-	-	-	−0.056	0.741	−0.047	0.823

Data are presented as the correlation coefficient.

4h-ICU, first four hours in the intensive care unit; CI_Max, the maximum value for cardiac index; CI_Min, the minimum value for cardiac index; CPB, cardiac pulmonary bypass; LCT_Max, the maximum value for lactate; LCT_Min, the minimum value for lactate; MAP_Max, the maximum value for mean arterial pressure; MAP_Max, the maximum value for mean arterial pressure; MAP_Min, the minimum value for mean arterial pressure; MPOC, major perioperative complications; Pre-CPB, preoperative cardiac pulmonary bypass; Post-CPB, postoperative cardiac pulmonary bypass; SvO_2__Max, the maximum value for mixed venous oxygen saturation; SvO_2__Min, the minimum value for mixed venous oxygen saturation; XL_Min, the minimum value for gastric reactance.

Table 5 summarizes the ROC analysis for key variables across different time points. Specifically, post-CPB, XL_Min demonstrated good predictive performance with an AUC of 0.73 (95% CI: 0.56–0.89), sensitivity of 0.65, and high specificity of 0.86. SvO2_Min showed even higher sensitivity at 0.85 and an AUC of 0.76 (95% CI: 0.58–0.93), reflecting strong discriminatory ability. Other variables, including LCT_Max, CI Min, and MAP Min, exhibited moderate predictive accuracy, with AUCs ranging from 0.65–0.68. Overall, these findings underscore XL_Min and SvO2_Min as the most reliable predictors of post-CPB morbimortality in this cohort.

**Table 5:** ROC curves analysis per time-event and variable.

Time-event	Measurement	ROC curve area (CI 95%)	Cut-off point	Sensitivity (CI 95%)	Specificity (CI 95%)	PPV (CI 95%)	NPV (CI 95%)
Preoperative							
Enrollment	EuroSCORE II (%)	0.55 (0.34, 0.75)	2.84	0.57 (0.3,1)	0.71 (0.14,1)	0.76 (0.64,0.94)	0.5 (0.41,1)
Enrollment	STS (%)	0.57 (0.28, 0.86)	0.64	1 (0.14,1)	0.38 (0.25,1)	0.74 (0.7,1)	1 (0.4,1)
Intraoperative							
Pre-CPB	XL Min (-jΩ)	0.66 (0.46, 0.85)	15.78	0.7 (0.52,0.91)	0.79 (0.43,1)	0.84 (0.7,1)	0.61 (0.48,0.85)
Pre-CPB	LCT_Max (mmol/L)	0.61 (0.40, 0.80)	1.65	0.39 (0.26,0.91)	0.93 (0.5,1)	0.9 (0.69,1)	0.48 (0.42,0.79)
Pre-CPB	SvO2 Min (%)	0.74 (0.57, 0.90)	72.5	0.7 (0.3,0.96)	0.71 (0.5,1)	0.8 (0.71,1)	0.59 (0.46,0.89)
Pre-CPB	CI Min (L/min/m^2^)	0.53 (0.32, 0.74)	1.46	0.55 (0.09,1)	0.62 (0.08,1)	0.71 (0.65,1)	0.44 (0.39,1)
Pre-CPB	MAP Min (mmHg)	0.45 (0.26, 0.64)	51.5	0.17 (0.04,0.91)	1 (0.29,1)	1 (0.67,1)	0.42 (0.39,0.67)
Post-CPB	XL Min (-jΩ)	0.73 (0.56, 0.89)	16.9	0.65 (0.39,0.87)	0.86 (0.71,1)	0.88 (0.8,1)	0.6 (0.48,0.79)
Post-CPB	LCT_Max (mmol/L)	0.65 (0.46, 0.83)	3.25	0.61 (0.26,0.91)	0.71 (0.43,1)	0.78 (0.69,1)	0.53 (0.43,0.83)
Post-CPB	SvO2 Min (%)	0.76 (0.58, 0.93)	77.5	0.85 (0.45,1)	0.57 (0.36,1)	0.74 (0.67,1)	0.73 (0.52,1)
Post-CPB	CI Min (L/min/m^2^)	0.65 (0.44, 0.87)	2.66	0.95 (0.35,1)	0.46 (0.23,1)	0.73 (0.67,1)	0.86 (0.5,1)
Post-CPB	MAP Min (mmHg)	0.68 (0.51, 0.86)	58.5	0.57 (0.26,0.96)	0.79 (0.43,1)	0.81 (0.7,1)	0.52 (0.44,0.85)
Postoperative							
ICU admission	SOFA (points)	0.62 (0.42, 0.81)	8.5	0.95 (0.1,1)	0.21 (0.14,1)	0.63 (0.61,1)	0.75 (0.44,1)
ICU admission	APACHE II (points)	0.62 (0.43, 0.82)	19.5	0.7 (0.2,0.95)	0.57 (0.36,1)	0.7 (0.62,1)	0.57 (0.44,0.85)
First 4 h in the ICU	XL Min (-jΩ)	0.47 (0.27, 0.67)	22.32	0.4 (0.05,0.75)	0.79 (0.5,1)	0.73 (0.61,1)	0.48 (0.42,0.65)
First 4 h in the ICU	LCT_Max (mmol/L)	0.61 (0.41, 0.80)	3.65	0.65 (0.15,0.95)	0.64 (0.36,1)	0.72 (0.65,1)	0.56 (0.45,0.88)
First 4 h in the ICU	SvO2 Min (%)	0.65 (0.45, 0.85)	58.5	0.35 (0.24,1)	0.92 (0.23,1)	0.86 (0.62,1)	0.52 (0.48,1)
First 4 h in the ICU	CI Min (L/min/m^2^)	0.66 (0.46, 0.86)	3.28	1 (0.24,1)	0.29 (0.21,1)	0.63 (0.61,1)	1 (0.52,1)
First 4 h in the ICU	MAP Min (mmHg)	0.71 (0,53, 0.88)	65.67	0.71 (0.33,1)	0.64 (0.36,1)	0.75 (0.68,1)	0.6 (0.48,1)

**Figure 4:**
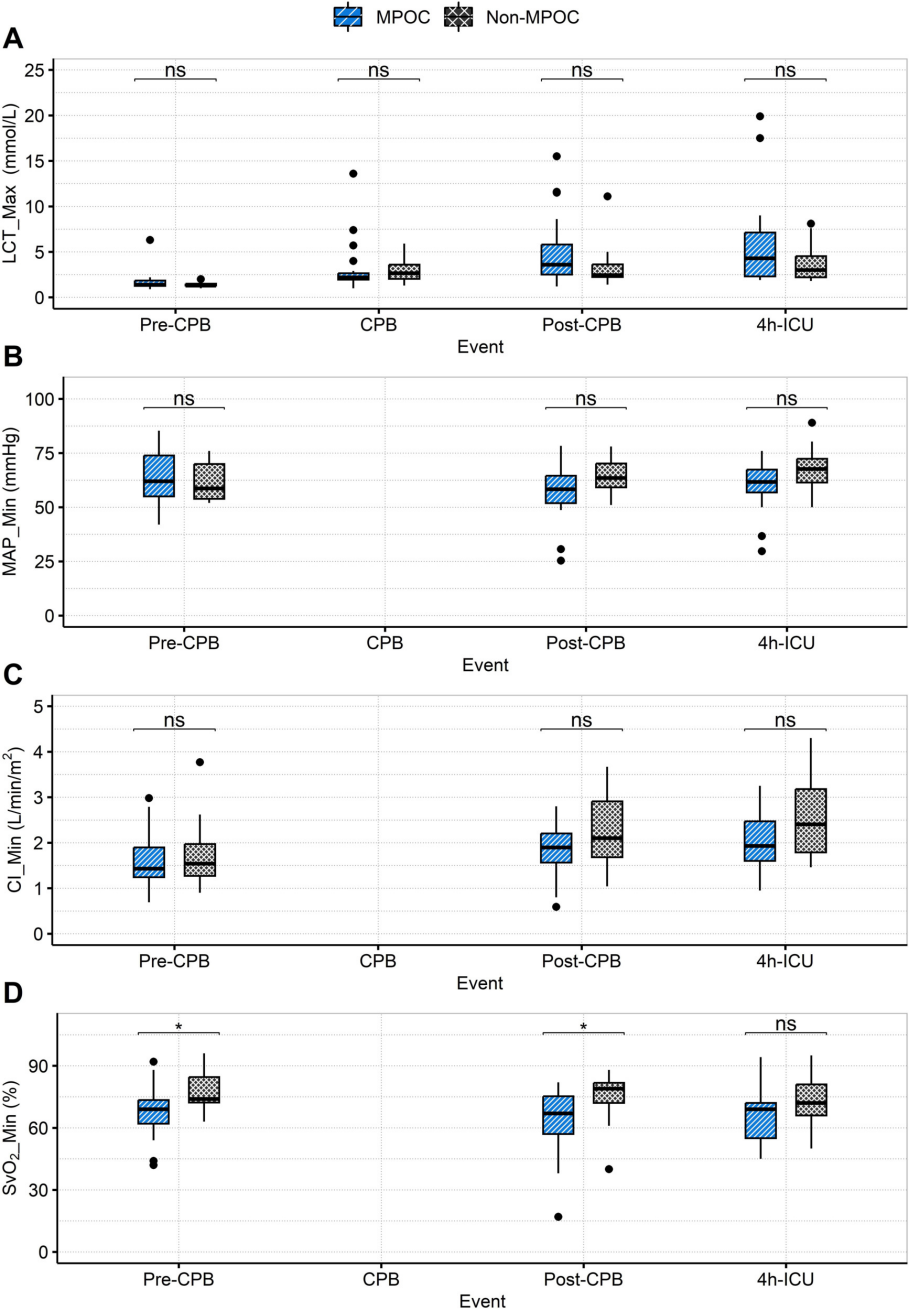
Intraoperative (pre-CPB, CPB, post-CPB) and postoperative (4h-ICU) event comparison for MPOC and Non-MPOC changes in **(A)** LCT_Max, **(B)** MAP_Min, **(C)** CI_Min and **(D)** SvO_2__Min. *for statistically significant results (*p* < 0.05). CI_Min, the minimum value for cardiac index; CPB, cardiopulmonary bypass; ICU, intensive care unit; LCT_Max, the maximum value for lactate; MAP_Min, the minimum value for mean arterial pressure; MPOC, major perioperative complications; ns, non-significant; Pre-CPB, preoperative cardiac pulmonary bypass; Post-CPB, postoperative cardiac pulmonary bypass; SvO2_Min, the minimum value for mixed venous oxygen saturation.

Supplementary Table S2 compares preoperative and postoperative laboratory test results, showing no statistically significant differences between groups. Supplementary Table S3 summarizes concomitant medication use, with vasopressin administration showing statistical significance only in the postoperative phase. The behavior of other variables is illustrated in Figure 4 and Supplementary Table S4, which presents the postoperative hemodynamic and oxygenation profiles.

**Figure 5:**
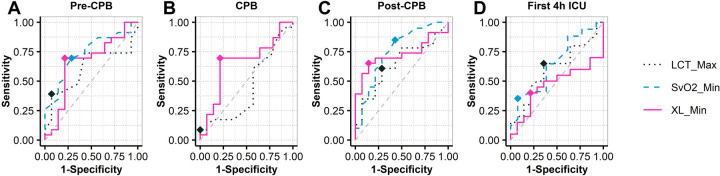
ROC analysis in XL_Min at intraoperative (pre-CPB, CPB, post-CPB) and postoperative (4h-ICU) events. CPB, cardiopulmonary bypass; ICU, intensive care unit; XL_Min, the minimum value for gastric reactance.

No adverse events related to the Florence monitor or Athena catheter were reported. Some unanticipated deviations were identified in clinical procedures, enrollment, and device utilization, prompting a protocol amendment to address these issues.

## Discussion

This study examined the behavior of XL_Min in the MPOC and Non-MPOC patient groups and compared it against key macrocirculatory variables throughout perioperative events (Pre-CPB, CPB, Post-CPB, 4h-ICU) in high-risk patients undergoing cardiac surgery with CPB.

The demographic and clinical characteristics of the study population (Tables 1, 2) confirm that both groups present high-risk factors, with no statistically significant differences between them. Moreover, Table 2 highlights that “CPB-Time” and “Aortic Cross-Clamp Time” exceeded 140 min in both groups, a factor known to be associated with an increased incidence of intraoperative and postoperative complications in cardiac surgery (30), further indicating that both groups consisted of high-risk patients (30).

In the postoperative phase (Table 2), upon ICU admission, the MPOC group exhibited a higher APACHE II score (21.5 points, corresponding to a 30% probability of hospital mortality) compared to the Non-MPOC group (18.5 points, 12% probability of hospital mortality). The SOFA score reflected an 80% probability of mortality in both groups (scores of 12.5 and 12.0, respectively). Heart failure with reduced ejection fraction (HFrEF, defined as LVEF <40%) was observed in both groups at the 24 h postoperative phase. Additionally, the MPOC group exhibited greater oliguria than the Non-MPOC group. Postoperative bleeding showed statistically significant differences between the groups [median (IQR), 1.0 (0.9) L, *p* < 0.001], indicating a higher mortality risk in the MPOC group (1).

The following sections discuss the relevant findings of key macrocirculatory variables and XL_Min across intraoperative and postoperative phases, as presented in Tables 3, 4, and Figure 3.

### Intraoperative phase

#### Pre-CPB

During this event, both groups exhibited clinical evidence of hemodynamic instability, with cardiac indices <2.2 L/min/m² and PAWP ≥ 15 mmHg, suggesting left ventricular dysfunction. MAP_Min indicated a tendency toward hypotension in both groups, although lactate levels remained within normal ranges, suggesting the absence of hypoperfusion, consistent with a SCAI-B classification (6). Additionally, in the MPOC group, SvO_2_ was <70%, indicating an emerging imbalance between oxygen supply and demand. The behavior of XL_Min during this event showed no statistically significant MLS between the groups.

#### CPB

During this event hemodynamic measurements using the PAC are not possible due to the use of CPB. However, both groups exhibited a tendency toward hyperlactatemia (≥2.0 mmol/L), indicating the onset of hypoperfusion. A MLS of 4.03 -jΩ in XL_Min values was observed in the MPOC group compared to the Non-MPOC group, although this location difference was not statistically significant.

#### Post-CPB

In this event, both groups exhibited persistent hyperlactatemia, with a greater increase observed in the MPOC group, which had a median (IQR) of 3.60 (3.30) mmol/L. Furthermore, the MPOC group demonstrated sustained hypotension, while the Non-MPOC group showed a tendency towards MAP >60 mmHg. The cardiac index in the MPOC group remained ≤2.2 L/min/m², while in the Non-MPOC group, it increased to a median (IQR) of 2.38 (1.95) L/min/m². Both groups presented PAWP > 15 mmHg. SvO_2_ values showed significant differences between the groups, with values <70% in the MPOC group, indicating that the majority of MPOC patients met the criteria for hypoperfusion, consistent with a SCAI-C (6). Notably, during this event, the MLS for XL_Min was 6.14 -jΩ higher in the MPOC group compared to the Non-MPOC group, showing statistical significance.

### Postoperative phase (4 h-ICU)

During the first 4 h in the ICU, both groups maintained hyperlactatemia, although only the MPOC group experienced episodes of mild hypotension. Cardiac indexes improved in both groups, with values exceeding 2.2 L/min/m², likely due to pharmacological management. In the Non-MPOC group, the maximum values reached a median (IQR) of 3.29 (1.57) L/min/m², with statistical significance, as well as SvO_2_ values of 81%. Moreover, the MPOC group continued to show signs of hypoperfusion. The MLS of XL_Min was −0,75 -jΩ, not showing statistical significance.

### XL_Min measurements

XL_Min in the MPOC group consistently presented higher values compared to the Non-MPOC group throughout all phases, indicating that complicated patients presented more severe hypoperfusion in the gastric wall, particularly at the post-CPB event where differences are statistically significant [MLS = 6.14 -jΩ, 95% CI (1.06, 11.34), *p* = 0.022*]. This significant location shift suggests that XL_Min serves as a sensitive marker of gastric mucosal ischemia during cardiac surgery with CPB. These results support the potential clinical utility of continuous monitoring XL_Min to detect early signs of splanchnic hypoperfusion, thereby enabling timely identification and intervention for patients at higher risk of complications starting from the intraoperative phase. The ROC analysis (see Figure 5) shows that XL_Min is potential predictor for post-CPB morbimortality, with an AUC of 0.73. Its combination of moderate sensitivity and high specificity suggests it can effectively identify patients at risk. These results point in the direction that XL_Min is a valuable marker for assessing patient prognosis after CPB. Compared to XL_Min, SvO2_Min demonstrated a slightly higher AUC of 0.76 and greater sensitivity, indicating stronger discriminatory power. Nonetheless, both variables appear to be valuable markers for assessing patient prognosis after CPB.

These findings, particularly at the Post-CPB phase, align with previous investigations examining the behavior of regional perfusion markers across different phases of CPB. Gastric tonometry measurements, for example, have been reported to demonstrate abnormally low values immediately after weaning from CPB, being recognized as a sensitive predictor of postoperative complications (31, 32). Similarly, microcirculation imaging studies have shown that the microcirculatory flow index (MFI) increases significantly at the conclusion of CPB in patients who subsequently develop complications (33). More recently, investigations of mitochondrial oxygen tension have indicated that patients who develop acute kidney injury (AKI) after cardiac surgery exhibit persistently reduced mitochondrial oxygen tension compared with those who do not develop AKI (34). These observed parallels underscore the need for further evaluation of the predictive value of XL, both in the setting of elective cardiac surgery and in other patient populations at elevated risk of hemodynamic instability.

Finally, it should be noted that Table 4 reveals that XL_Min has no clinical association with macrocirculatory variables and risk scores. Such a discrepancy might be explained by the phenomenon of hemodynamic incoherence that occurs when macrocirculatory markers appear normal, but microcirculatory perfusion is impaired (35–37). This phenomenon is frequently observed during CPB, where systemic inflammatory responses, endothelial activation, and altered rheology disrupt the alignment between global and regional circulation (38). An important limitation of the present study is the absence of direct microcirculatory imaging, which would have allowed to study the association between XL and microvascular dysfunction; nevertheless, the present findings reinforce the importance of incorporating microcirculatory assessment into future investigations as, advancing the integration of microvascular endpoints into perioperative hemodynamic management has been increasingly recognized as a pathway to more personalized and effective care (39).

Concordance between our findings and prior gastric tonometry and microcirculation imaging studies, in which abnormal measurements immediately after weaning from CPB predict postoperative complications, suggests that XL may provide an early signal of splanchnic compromise. It may also help with early assessment of increased gut barrier permeability and the attendant risk of endotoxemia, consistent with earlier advocacy for gastric tonometry (40). These observations are specific to the population studied and should not be generalized without caution. At the same time, the physiological determinants of XL and their link to the complications observed in this study remain incompletely characterized, and a direct mechanistic link between microcirculatory alterations and XL has not been demonstrated. Prospective studies are needed to test its association with direct microcirculatory measurements, to define clinically actionable thresholds, and to quantify incremental predictive value over standard monitoring using patient-centered outcomes. The potential value of XL at this stage is as a complementary marker for perioperative risk assessment in cardiac surgery rather than as a replacement for macrocirculatory variables.

### Limitations

The selection criteria for the study focused on high-risk patients undergoing CPB at a single site, resulting in a modest sample size that may be affected by selection bias [the lower bound for the sample size according to the methodology proposed by Peduzzi and Concato (27) is 34 and the study included 37 patients]. Consequently, the statistical significance of XL_Min might be different in other clinical settings, lower-risk patients, or patients undergoing beating-heart procedures.

On the other hand, the lack of clinical association between XL_Min and macrocirculatory variables suggest that XL_Min may open an avenue for further understanding the phenomenon of hemodynamic incoherence, also noticed in the context of microcirculation imaging and urethral photoplethysmography (11, 13); however, the study didn't include validated microcirculation markers that could shed light on their correlation with XL_Min.

Finally, the bedside monitor (Florence, model ISMO 1.0, Alandra Medical) is provided with algorithms that automatically discard unreliable measurements caused by external factors such as movement artifacts, enteral feeding, or electromagnetic interference emitted by electrosurgery units, or transesophageal echocardiography devices. Discarding such measurements resulted in unequal sampling of the XL signal during such events.

## Conclusion

In high-risk cardiac surgery patients, XL_Min values were consistently higher in those who developed MPOC, with statistically significant differences observed in the post-CPB phase. These findings suggest that gastric impedance spectroscopy may aid in the early identification of patients at risk of MPOC by detecting gastric mucosal hypoperfusion. The technique proved safe and feasible for intraoperative and postoperative monitoring.

## Data Availability

The raw data supporting the conclusions of this article will be made available by the authors, without undue reservation.

## References

[1] Stephens RS Whitman GJR. Postoperative critical care of the adult cardiac surgical patient: part II: procedure-specific considerations, management of complications, and quality improvement. Crit Care Med. (2015) 43(9):1995–2014. 10.1097/CCM.0000000000001171 26136101

[2] Hendy A Bubenek-Turconi ŞI. The diagnosis and hemodynamic monitoring of circulatory shock: current and future trends. J Crit Care Med. (2016) 2(3):115–23. 10.1515/jccm-2016-0018 PMC5953246 29967849

[3] Schwarzova K Damle S Sellke FW Robich MP. Gastrointestinal complications after cardiac surgery. Trauma Surg Acute Care Open. (2024) 9(1):e001324. 10.1136/tsaco-2023-001324 38616788 PMC11015217

[4] Schoe A Bakhshi-Raiez F De Keizer N Van Dissel JT De Jonge E. Mortality prediction by SOFA score in ICU-patients after cardiac surgery; comparison with traditional prognostic-models. BMC Anesthesiol. (2020) 20(1):1–8. 10.1186/s12871-020-00975-2 32169047 PMC7068937

[5] Sinha S Dimagli A Dixon L Gaudino M Caputo M Vohra HA Systematic review and meta-analysis of mortality risk prediction models in adult cardiac surgery. Interact Cardiovasc Thorac Surg. (2021) 33(5):673–86. 10.1093/icvts/ivab151 34041539 PMC8557799

[6] Naidu SS Baran DA Jentzer JC Hollenberg SM van Diepen S Basir MB SCAI SHOCK stage classification expert consensus update: a review and incorporation of validation studies: this statement was endorsed by the American college of cardiology (ACC), American college of emergency physicians (ACEP), American heart association (AHA), European society of cardiology (ESC) association for acute cardiovascular care (ACVC), international society for heart and lung transplantation (ISHLT), society of critical care medicine (SCCM), and society of thoracic surgeons (STS) in december. J Am Coll Cardiol. (2022) 79(9):933–46. 10.1016/j.jacc.2022.01.018 35115207

[7] Bootsma IT Boerma EC Scheeren TWLL de Lange F. The contemporary pulmonary artery catheter. Part 2: measurements, limitations, and clinical applications. J Clin Monit Comput. (2021) 36(0123456789):17–31. 10.1007/s10877-021-00673-5 33646499 PMC7917533

[8] Rajkumar KP Hicks MH Marchant B Khanna AK. Blood pressure goals in critically ill patients. Methodist Debakey Cardiovasc J. (2023) 19(4):24–37. 10.14797/mdcvj.1260 37547901 PMC10402811

[9] Fuller BM Dellinger RP. Lactate as a hemodynamic marker in the critically ill. Curr Opin Crit Care. (2012) 18(3):267–72. 10.1097/MCC.0b013e3283532b8a 22517402 PMC3608508

[10] Minton J Sidebotham DA. Hyperlactatemia and cardiac surgery. J Extra Corpor Technol. (2017) 49(1):7–15. 10.1051/ject/201749007 28298660 PMC5347225

[11] Flick M Hilty MP Duranteau J Saugel B. The microcirculation in perioperative medicine: a narrative review. Br J Anaesth. (2024) 132(1):25–34. 10.1016/j.bja.2023.10.033 38030549

[12] Harms FA Streng LWJM Wefers Bettink MA de Wijs CJ Römers LH Janse R Monitoring of mitochondrial oxygen tension in the operating theatre: an observational study with the novel COMET® monitor. PLoS One. (2023) 18(2):e0278561. 10.1371/journal.pone.0278561 36758026 PMC9910761

[13] Flick M Rosenau L Sadtler H Kouz K Krause L Joosten A The urethral perfusion index during off-pump coronary artery bypass surgery: an observational study. J Cardiothorac Vasc Anesth. (2024) 38(2):417–22. 10.1053/j.jvca.2023.09.015 38114369

[14] Kopp R Dommann K Rossaint R Schälte G Grottke O Spillner J Tissue oxygen saturation as an early indicator of delayed lactate clearance after cardiac surgery: a prospective observational study. BMC Anesthesiol. (2015) 15(1):158. 10.1186/s12871-015-0140-7 26518485 PMC4628313

[15] Godinez-Garcia MM Soto-Mota A Catrip J Gaitan R Lespron MdC Molina FJ Comparison of gastric reactance with commonly used perfusion markers in a swine hypovolemic shock model. Intensive Care Med Exp. (2022) 10(1):1–16. 10.1186/s40635-022-00476-1 36400981 PMC9674824

[16] Beltran NE Sacristan E. Gastrointestinal ischemia monitoring through impedance spectroscopy as a tool for the management of the critically ill. Exp Biol Med (Maywood). (2015) 240(7):835–45. 10.1177/1535370215571876 25711880 PMC4935395

[17] Peña-Mercado E Garcia-Lorenzana M Patiño-Morales CC Montecillo Aguado M Huerta Yepez S Beltran NE. Bioelectric, tissue, and molecular characteristics of the gastric mucosa at different times of ischemia. Exp Biol Med. (2021) 246(18):1968–80. 10.1177/15353702211021601 PMC8474982 34130514

[18] Peña-Mercado E Garcia-Lorenzana M Huerta-Yepez S Cruz-Ledesma A Beltran-Vargas NE. Effect of melatonin on electrical impedance and biomarkers of damage in a gastric ischemia/reperfusion model. PLoS One. (2022) 17(8):e0273099. 10.1371/journal.pone.0273099 35972989 PMC9380938

[19] McGee WT Young C Fraizer JA, editors. Edwards Clinical Education: Quick Guide to Cardiopulmonary Care. 4th ed. Irvine, CA: Edwards Lifesciences (2018). p. 163–5.

[20] Murphy GS Hessel EA Groom RC. Optimal perfusion during cardiopulmonary bypass: an evidence-based approach. Anesth Analg. (2009) 108(5):1394–417. 10.1213/ane.0b013e3181875e2e 19372313

[21] Hessel EA. What’s new in cardiopulmonary bypass. J Cardiothorac Vasc Anesth. (2019) 33(8):2296–326. 10.1053/j.jvca.2019.01.039 30928282

[22] Barry AE Chaney MA London MJ. Anesthetic management during cardiopulmonary bypass: a systematic review. Anesth Analg. (2015) 120(4):749–69. 10.1213/ANE.0000000000000612 25790208

[23] Rauch G Schüler S Kieser M. Planning and Analyzing Clinical Trials with Composite Endpoints (2017).

[24] Food and Drug Administration. Multiple Endpoints in Clinical Trials: Guidance for Industry. Guidance Document. Rockville, MD: Food and Drug Administration (2022). p. 1–26.

[25] Dyke C Aronson S Dietrich W Hofmann A Karkouti K Levi M Universal definition of perioperative bleeding in adult cardiac surgery. J Thorac Cardiovasc Surg. (2014) 147(5):1458–1463.e1. 10.1016/j.jtcvs.2013.10.070 24332097

[26] Levy B Fritz C Tahon E Jacquot A Auchet T Kimmoun A. Vasoplegia treatments: the past, the present, and the future. Crit Care. (2018) 22(1):52. 10.1186/s13054-018-1967-3 29486781 PMC6389278

[27] Peduzzi P Concato J Kemper E Holford TR Feinstein AR. A simulation study of the number of events per variable in logistic regression analysis. J Clin Epidemiol. (1996) 49(12):1373–9. 10.1016/S0895-4356(96)00236-3 8970487

[28] Madley-Dowd P Hughes R Tilling K Heron J. The proportion of missing data should not be used to guide decisions on multiple imputation. J Clin Epidemiol. (2019) 110:63–73. 10.1016/j.jclinepi.2019.02.016 30878639 PMC6547017

[29] Churpek MM Adhikari R Edelson DP. The value of vital sign trends for detecting clinical deterioration on the wards. Resuscitation. (2016) 102:1–5. 10.1016/j.resuscitation.2016.02.005 26898412 PMC4834231

[30] Jucá FG Freitas Fd Goncharov M Pes DdL Jucá MEC Dallan LRP Difference between cardiopulmonary bypass time and aortic cross-clamping time as a predictor of complications after coronary artery bypass grafting. Braz J Cardiovasc Surg. (2024) 39(2):e20230104. 10.21470/1678-9741-2023-0104 38426431 PMC10903005

[31] Landow L Phillips DA Heard SO Prevost D Vandersalm TJ Fink MP. Gastric tonometry and venous oximetry in cardiac surgery patients. Crit Care Med. (1991) 19(10):1226–33. 10.1097/00003246-199110000-00003 1914478

[32] Fiddian-Green RG Baker S. Predictive value of the stomach wall pH for complications after cardiac operations: comparison with other monitoring. Crit Care Med. (1987) 15(2):153–6. 10.1097/00003246-198702000-00015 3100137

[33] Prestes I Riva J Bouchacourt JP Kohn E López A Hurtado FJ. Alteraciones microcirculatorias en cirugía cardíaca con circulación extracorpórea. Rev Esp Anestesiol Reanim. (2016) 63(9):513–8. 10.1016/j.redar.2016.03.005 27095670

[34] Hilderink BN Crane RF Arbous SM van den Bogaard B Pillay J Juffermans NP. Low postoperative mitochondrial oxygen tension is an early marker of acute kidney injury after cardiac surgery: a prospective observational study. J Crit Care. (2025) 88:155088. 10.1016/j.jcrc.2025.155088 40267552

[35] Vincent JL Taccone FS. Microvascular monitoring—do ‘global’ markers help? Best Pract Res Clin Anaesthesiol. (2016) 30(4):399–405. 10.1016/j.bpa.2016.10.006 27931643

[36] Bakker J. Lactate levels and hemodynamic coherence in acute circulatory failure. Best Pract Res Clin Anaesthesiol. (2016) 30(4):523–30. 10.1016/j.bpa.2016.11.001 27931655

[37] Ince C Ertmer C. Hemodynamic coherence: its meaning in perioperative and intensive care medicine. Best Pract Res Clin Anaesthesiol. (2016) 30(4):395–7. 10.1016/j.bpa.2016.11.004 27931642

[38] De Cuyper H Poelaert J. Microcirculatory alterations in cardiac surgery: a comprehensive guide. J Cardiothorac Vasc Anesth. (2024) 38(3):829–38. 10.1053/j.jvca.2023.11.042 38195271

[39] Putowski Z Bakker J Kattan E Hernández G Ait-Oufella H Szczeklik W Tissue perfusion as the ultimate target of hemodynamic interventions in the perioperative period. J Clin Anesth. (2025) 107:112009. 10.1016/j.jclinane.2025.112009 40972268

[40] Lorusso R Mariscalco G Vizzardi E Bonadei I Renzulli A Gelsomino S. Acute bowel ischemia after heart operations. Annf Thorac Surg. (2014) 97(6):2219–27. 10.1016/j.athoracsur.2014.01.029 24681032

